# NCS-Rapgef2, the Protein Product of the Neuronal *Rapgef2* Gene, Is a Specific Activator of D1 Dopamine Receptor-Dependent ERK Phosphorylation in Mouse Brain

**DOI:** 10.1523/ENEURO.0248-17.2017

**Published:** 2017-09-25

**Authors:** Sunny Zhihong Jiang, Wenqin Xu, Andrew C. Emery, Charles R. Gerfen, Maribeth V. Eiden, Lee E. Eiden

**Affiliations:** 1 Section on Molecular Neuroscience, National Institute of Mental Health Intramural Research Program, Bethesda, MD 20892; 2 Section on Directed Gene Transfer Laboratory of Cellular and Molecular Regulation, National Institute of Mental Health Intramural Research Program, Bethesda, MD 20892; 3 Laboratory of Systems Neuroscience, National Institute of Mental Health Intramural Research Program, Bethesda, MD 20892

**Keywords:** amphetamine, cAMP, cell signaling, cocaine, GPCR, MAP kinase, psycho-stimulants

## Abstract

The neuritogenic cAMP sensor (NCS), encoded by the *Rapgef2* gene, links cAMP elevation to activation of extracellular signal-regulated kinase (ERK) in neurons and neuroendocrine cells. Transducing human embryonic kidney (HEK)293 cells, which do not express Rapgef2 protein or respond to cAMP with ERK phosphorylation, with a vector encoding a *Rapgef2* cDNA reconstituted cAMP-dependent ERK activation. Mutation of a single residue in the cyclic nucleotide-binding domain (CNBD) conserved across cAMP-binding proteins abrogated cAMP-ERK coupling, while deletion of the CNBD altogether resulted in constitutive ERK activation. Two types of mRNA are transcribed from *Rapgef2 in vivo*. Rapgef2 protein expression was limited to tissues, i.e., neuronal and endocrine, expressing the second type of mRNA, initiated exclusively from an alternative first exon called here exon 1’, and an alternative 5’ protein sequence leader fused to a common remaining open reading frame, which is termed here NCS-Rapgef2. In the male mouse brain, NCS-Rapgef2 is prominently expressed in corticolimbic excitatory neurons, and striatal medium spiny neurons (MSNs). Rapgef2-dependent ERK activation by the dopamine D1 agonist SKF81297 occurred in neuroendocrine neuroscreen-1 (NS-1) cells expressing the human D1 receptor and was abolished by deletion of *Rapgef2*. Corticolimbic [e.g., dentate gyrus (DG), basolateral amygdala (BLA)] ERK phosphorylation induced by SKF81297 was significantly attenuated in *CamK2α-Cre^+/−^*; *Rapgef2^cko/cko^* male mice. ERK phosphorylation in nucleus accumbens (NAc) MSNs induced by treatment with SKF81297, or the psychostimulants cocaine or amphetamine, was abolished in male *Rapgef2^cko/cko^* mice with NAc NCS-Rapgef2-depleting AAV-Synapsin-Cre injections. We conclude that D1-dependent ERK phosphorylation in mouse brain requires NCS-Rapgef2 expression.

## Visual Abstract

**Figure:**
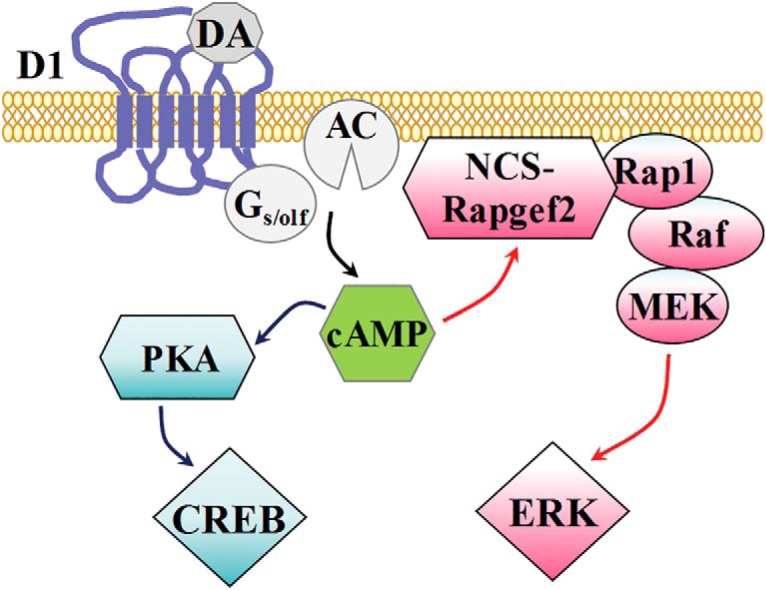

## Significance Statement

Our report demonstrates that the cAMP-regulated guanine nucleotide exchange factor neuritogenic cAMP sensor (NCS)-Rapgef2 is required for D1 receptor-dependent dopaminergic activation of the MAP kinase extracellular signal-regulated kinase (ERK) in neuroendocrine cells in culture, and in dopamine-innervated, D1 receptor-expressing regions of the adult mouse brain, including the hippocampus, amygdala and ventral striatum. NCS-Rapgef2 is the protein product of a neuronal/endocrine-specific set of mRNAs transcribed from an alternative promoter within the *Rapgef2* gene locus in both rodents and humans. NCS-Rapgef2 expression in nucleus accumbens (NAc) is required for ERK activation in this dopaminergic reward center by the psychostimulants cocaine or amphetamine, suggesting a role for NCS-Rapgef2 in the mechanisms of action of these drugs of abuse.

## Introduction

In a search for the cAMP sensor mediating Gs-coupled GPCR activation of neurite extension (neuritogenesis), we identified neuritogenic cAMP sensor (NCS)-Rapgef2 as a cAMP-dependent guanine nucleotide exchange factor mediating extracellular signal-regulated kinase (ERK) activation in neuronal and endocrine cells (Emery and Eiden, 2012; Emery et al., 2013). NCS-Rapgef2 is encoded within the *Rapgef2* gene locus, previously associated with transcription of putative proteins variously called KIAA0313, nRAP-GEP, PDZ-GEF1, and CNrasGEF, and variously associated with activation of Ras or Rap, by either cAMP, cGMP, or constitutively, in either neuronal or non-neuronal cells, during either adulthood or during development (Pham et al., 2000; Dremier et al., 2003; Kuiperij et al., 2003; Kuiperij et al., 2006; Kannan et al., 2007). Given the bewildering pleiotropy of the presumed function of Rapgef2 in cell signaling, yet its manifestly important role in ERK activation, we wished to ask the following three key questions relevant to Rapgef2 function, specifically in neuronal signaling. First, is Rapgef2 a cAMP-responsive protein in the context of the cells in which it is normally expressed? Second, with the reported pattern of expression of Rapgef mRNA and protein ranging from neuron-specific (Ohtsuka et al., 1999) to ubiquitous in most cell types (de Rooij et al., 1999) in the literature, could we define clearly, in the adult mouse, the pattern of expression of each of the Rapgef2 mRNA variants, and their relationships to protein expression in neuronal and non-neuronal tissues? Finally, with dominant non-neuronal and neuronal roles in development, and across multiple organ systems in organisms as diverse as mice and fruit flies, could neuron-specific Rapgef2 expression in the brain be traced to any clearly defined role in neurotransmission? Recent data implicating the *Rapgef2* gene locus in schizophrenia, as one of only nine copy number variations segregating to all genotyped affected members within several families with schizophrenia (Xu et al., 2009), and our own work implicating Rapgef2 in neuroendocrine control of ERK activation, a key component in cellular processes underlying learning and memory (Morozov et al., 2003), impelled a detailed investigation of the role of Rapgef2 in ERK neuronal signaling in the adult brain.

We have focused on dopamine-dependent activation of ERK, to answer these three questions, because of its importance in learning, memory and addiction in the mammalian brain (Berhow et al., 1996; Greengard et al., 1999; Valjent et al., 2000; Mizoguchi et al., 2004; Kamei et al., 2006). The D1 dopamine receptor on neurons postsynaptic to dopaminergic inputs responds to dopamine occupancy by Gs/Golf-dependent activation of adenylate cyclase, and elevation of cAMP (Beaulieu and Gainetdinov, 2011). Protein kinase A (PKA) is the best characterized neuronal cAMP sensor, but others have been identified. These include the exchange proteins activated by cAMP (Epacs; de Rooij et al., 1998; Kawasaki et al., 1998) and NCS-Rapgef2 (Emery et al., 2013; Emery et al., 2014).

Pharmacological and genetic analyses have shown that dopamine-dependent ERK regulation occurs primarily via the D1 dopamine receptor (Valjent et al., 2000; Zhang et al., 2004; Valjent et al., 2005; Gerfen et al., 2008). D1-dependent ERK activation has been shown to underlie the effects of psychomotor stimulants on behaviors including enhanced locomotor activity, and drug-seeking behaviors seen after chronic exposure (Pierce et al., 1999; Valjent et al., 2000; Cahill et al., 2014). In the striatum, a D1 receptor-dependent signaling pathway that indirectly enhances ERK activity via PKA has been characterized in detail (Greengard et al., 1999; Valjent et al., 2005). However, direct activation of ERK through D1-dependent cAMP activation may also occur in dopaminoceptive neurons in the brain (Gerfen et al., 2002; Gerfen et al., 2008; Fasano et al., 2009; Nagai et al., 2016). In this report, we demonstrate that Rapgef2 mediates D1-dependent ERK activation in neurons of the adult hippocampus, amygdala, and ventral striatum of mice.

## Materials and Methods

### Drugs and reagents

8-(4-chlorophenylthio) adenosine-3’,5’-cyclic monophosphate (8-CPT-cAMP) was purchased from BIOLOG Life Science Institute. The inhibitors 9-(tetrahydro-2-furanyl)-9H-purin-6-amine (SQ22536), 1,4-diamino-2,3-dicyano-1,4-bis[2-aminophenylthio]butadiene (U0126), N-[2-[[3-(4-bromophenyl)-2-propenyl-]amino]ethyl]-5-isoquinolinesulfonamide dihydrochloride (H-89), and α-[amino[(4-aminophenyl)thio]methylene]-2-(trifluoromethyl)benzeneacetonitrile (SL327), and D1 receptor ligands SKF81297 hydrobromide and SCH-23390 hydrochloride were purchased from Tocris. Reserpine and phorbol 12-myristate 13-acetate (PMA) were obtained from Sigma. Drug stocks were prepared at 50 or 100 mM in DMSO, followed by dilution in culture media to final concentrations of 100 μM 8-CPT-cAMP, 10 μM reserpine, 10 μM U0126, 30 µM H89, 1 mM SQ22536, 10 µM SCH-23390, 10 µM SKF 81297, and 100 nM PMA.

### Cell culture

All solutions used for cell culture were purchased from Life Technologies unless otherwise noted. Neuroscreen-1 (NS-1) cells are a subclone of PC12 cells purchased from Cellomics. NS-1 cells were cultured in Roswell Park Memorial Institute (RPMI) 1640 medium supplemented with 10% horse serum (HyClone), 5% heat-inactivated fetal bovine serum, 2 mM L-glutamine, 100 U/ml penicillin, and 100 µg/ml streptomycin. Human embryonic kidney (HEK)293 cells and human neuroblastoma SH-SY5Y (obtained from ATCC) were grown in DMEM supplemented with 10% FBS (Atlanta Biologicals), 2 mM l-glutamine, 100 U/ml penicillin, and 100 μg/ml streptomycin. Cells were grown in flasks (Techno Plastic Products) coated with collagen type I from rat tail as described previously (Hamada et al., 2012) at 37°C in a humidified incubator containing 5% CO_2_.

### Establishment of stable cell lines

Human *Rapgef2* cDNA (*hRapgef2*) was obtained from Open Biosystems as a MGC clone (catalog MHS4426-99239299). The *hRapgef2* cDNA was first cloned into pPRIChp retroviral vector (Albagli-Curiel et al., 2007) using Not1 and BstB1 restriction sites. Rapgef2 mutant (pRapGEF2 aa-Δ14) with deleted cAMP binding site (residues 124-238) was generated by removing residues 2-242 from the Rapgef2 protein using the QuikChange Site-Directed Mutagenesis kit (Stratagene) according to the manufacturer’s instructions, with complementary primers including sense primer (5’-CTTGCAATCCCAGCTAACCATGGAGTTATGAAAGAACACCGAGGAACTTG-ATCGAACTGG-3’) and its reverse complement as antisense primer. Another Rapgef2 mutant named pRapgef2-K211D within which the cyclic nucleotide-binding domain (CNBD)-conserved lysine residue at position 199 was converted to an aspartic acid residue was similarly constructed using mutagenesis with sense primer (5’-GGACAAAGAATACATGGACGGAGTGATGAGAACAAAGG-3’ and antisense 5’-CCTTTGTTCTCATCACTCCGTCCATGTATTCTTTGTCC-3’). cDNA fragments encoding hRapgef2 and mutants were transferred into a pPiggyBac vector (Pb511) obtained from System Biosciences using NheI and BamH1 restriction sites to make pPB-hRapgef2, pPB-pRapgef2 aa-Δ14 and pPB-hRapgef2-K211D. The stable cell line HEK293-Cre-Luc [HEK293 stably expressing a cAMP-response element (CRE)-driven luciferase reporter gene], described previously (Emery et al., 2016), was seeded at a density of 5 × 10^5^/well in a six-well plate the night before DNA transfection with plasmids encoding pPB-hRapgef2 (or individual mutant) and PiggyBac transposase at a ratio of 3:1. A total of 2.5 µg of total DNA was transfected into each well using Lipofectamine 3000 (Thermo Fisher Scientific). After 48 h, cells were selected with G418 for two weeks before being employed for experiments involving functional analysis of hRapgef2. A stable NS-1 cell line expressing the human D1 receptor was generated using the pL304 lentiviral vector encoding hDRD1(GenBank accession number BC074978.2) and the blasticidin resistance gene bsr (GeneCopoeia). pVSV-G env, pLenti gag/pol/rev, and pL304-hDRD1 plasmids were transfected into HEK293T cells using a Profection kit (Promega). Viral supernatants were collected 48 h after transfection, filtered, and used to transduce NS-1 cells. Forty-eight hours after transduction, cells were subject to 3 µg/ml blasticidin selection for two weeks to generate NS-1 cells stably expressing DRD1. NS1 cells stably expressing Cas9 nuclease were generated using lentiCas9-Blast (Addgene plasmid 52962; Sanjana et al., 2014) lentiviral vector followed by blasticidin selection. CrRNAs against the cAMP binding site of *hRapgef2* gene were purchased from Integrated DNA Technologies (IDT). The delivery of crRNAs into NS1Cas9 cells and detection of gene editing was performing according to IDT Alt-R CRISPR-Cas9 user guide. After gene editing, individual cell clones were obtained by serial dilution. The region of hRapgef2 cAMP binding site was PCR cloned from genomic DNA of each cell clone and sequenced to confirm of the biallelic deletion of this region. Cell lines with confirmed deletion were then used for neuritogenesis assay.

### Neuritogenesis and cell growth assays

NS-1 cells removed from antibiotic selection were dispensed into six-well plates, and the following day, vehicle or pharmacological agents were added in fresh medium. Cells were treated for 48 or 72 h, as indicated. Using a 20× objective, photomicrographs were then acquired with a computer-assisted microscope. Images were randomized and a blinded observer counted the number of cells, the number of neurites, and the length of the neurites in each field using NIS-Elements BR (Nikon).

### Western blotting for cultured cell and mouse tissues

Cells were seeded in 12-well plates and collected in ice-cold lysis buffer (150 mM NaCl, 50 mM Tris-HCl, 1% NP-40, and 1 mM EDTA) containing 1× Halt Protease and Phosphatase Inhibitor Cocktail (Thermo Scientific, catalog 78440). Tissues were dissected, weighed, and snap frozen. Samples were solubilized in ice-cold RIPA buffer (25 mM Tris-HCl pH 7.4, 150 mM NaCl, 1% NP-40, 1% sodium deoxycholate, and 0.1% SDS) supplemented with 5 mM EDTA and 4× Halt Protease and Phosphatase Inhibitor Cocktail (Thermo Scientific, catalog 78440). For every 10 mg of tissue (wet weight), 300 μl of lysis buffer was applied, and samples were sonicated on ice. Following sonication, RIPA-insoluble fractions were removed by centrifugation (4000 rpm for 10 min at 4°C). Supernatants were retained, and protein concentration was determined by a colorimetric bicinchoninic acid-based protein assay (Thermo Scientific, catalog 23225) conducted according to the protocol provided by the manufacturer. Equivalent amounts of protein for each sample were mixed with loading buffer and separated by SDS-PAGE on 4-12% polyacrylamide Bis-Tris gels (Invitrogen) or Bullet PAGE One Precast Gels with a 5-11% polyacrylamide gradient (Nacalai catalog 13077-04). Gels were blotted onto nitrocellulose membranes (Invitrogen) using a semi-dry transfer apparatus (Invitrogen) at 30 V for 2 h at room temperature. Membranes were then blocked with 5% skim milk dissolved in Tris-buffered saline with 1% Tween 20 (TBST) for 2 h, incubated overnight at 4°C with rabbit anti-Rapgef2 (NNLE-2; 1:1000 in TBST with 5% BSA), washed three times in TBST, and incubated with appropriate HRP-coupled secondary antibodies (Cell Signaling Technology, catalog 7074) in blocking buffer for 1 h. Membranes were washed three times for 15 min in TBST and once in PBS. Immunoreactive bands were visualized with a chemiluminescent substrate (Thermo Scientific, catalog 34078) and photographed with a cooled charged-coupled device camera (Protein Simple). The NNLE-2 antibody used in these experiments was custom-made against an N-terminal epitope of Rapgef2 (LPADFTKLHLTDSLH), which is downstream of alternatively encoded N-terminal protein variants (i.e., within exon 2). The peptide was synthesized with an N-terminal cysteine residue, conjugated to keyhole limpet haemocyanin, and injected into New Zealand white rabbits in a standard immunization protocol. Serum from a single rabbit was affinity purified by Anaspec.

### RNA extraction and PCR characterization of Rapgef2-related transcripts

Mouse total RNA from different tissues was purchased from Clontech. Total RNA extractions from cell lines were performed using RNAqueous kit (Thermo Fisher Scientific) following the manufacturer’s manual. RNA samples were subject to Turbo DNase I (Thermo Fisher Scientific) digestion before cDNA synthesis. Total RNA (2 µg) was reverse transcribed using SuperScript III First-Strand Synthesis System (Thermo Fisher Scientific). To amplify each specific *Rapgef2* transcript from cDNA, PCR was preformed using the Takara PrimeSTAR HS DNA polymerase (Takara). Primers are listed in Extended Data Figure 1-2. Sense primers are specific to the individual exon 1 or exon 1’, antisense primers are complementary to sequences spanning exon 2/3 boundary. PCR products were gel purified and cloned into a TA vector using TA PCR cloning kit (Thermo Fisher). Sequences were verified by sequencing analysis.

### Cell-based ELISA for phosphorylated ERK

Phosphorylated ERK was measured according to a protocol described previously (Versteeg et al., 2000). Stable HEK293 cell lines were seeded at density of 3 × 10^5^ per well in 96-well plates and grown overnight. Cells were treated for 30 min followed by removal of media and fixation in 4% formaldehyde in PBS for 20 min at room temperature. Fixed cells were permeabilized by three washes in 0.1% Triton X-100 in PBS (PBST), and endogenous peroxidase activity was quenched by 20-min incubation in PBS containing 0.6% H_2_O_2_. Following three washes in PBST, samples were blocked (10% FBS in PBST) for 1 h and incubated overnight with primary antibodies against phospho-ERK1/2 (Cell Signaling Technology #9101) at 1:500. Samples were washed three times in PBST and twice in PBS. An HRP-coupled anti-rabbit secondary antibody (CST #7074), diluted 1:500 in PBST containing 5% BSA, was added for 1 h at room temperature. Following five washes in PBST, samples were exposed to the colorimetric substrate One-Step Ultra TMB-ELISA (Pierce, catalog 34028). After development in the dark for 10 min, the reaction was stopped by adding 4 M sulfuric acid, and absorbance was read at 450 nm.

### Animals and drug treatments

Male mice (wild type or transgenic) on C57BL6N background were housed two to five per cage and acclimatized to a 12/12 h light/dark cycle with food and water *ad libitum*. Animal care, drug treatment and surgeries were approved by the NIMH Institutional Animal Care and Use Committee and conducted in accordance with the NIH guidelines. The floxed Rapgef2 mouse strain (*Rapgef2^cko/cko^*) was a gift from Dr. Steven Hou, National Cancer Institute. It was indicated in the original paper (Satyanarayana et al., 2010) that Rapgef2 exon 18 was targeted and flanked by two loxP sites to make *Rapgef2^cko/cko^* mice. However, by sequence blast of primers for genotyping and subcloning of the sequence between two loxP sites, we found that Rapgef2 conditional targeting area is exon 4, which encodes cAMP binding domain. Subcloning and sequencing of *Rapgef2 cKO* allele were conducted as below: genomic DNA of wild-type mice and *Rapgef2^cko/cko^* mice was extracted using genomic DNA purification kit (Qiagen, MD). A total of 200 ng of each DNA was used as template for PCR analysis using PrimeStar DNA polymerase following the manufacturer’s instruction. Sense primer: 5’-gtggcagcagaggatcgc-3’, and antisense primer 5’-ccacagccgtctgagcag-3’ complementary to sequences flanking mouse *Rapgef2* exon 3 to exon 5 were used to amplify fragments including exons 3, 4, and 5. The PCR product was subcloned into TOPO cloning vector pCR2.1 and subject to sequence analysis. *Camk2α-cre* (*T29-1*) transgenic mice were purchased from The Jackson Laboratory and crossed with *Rapgef2^cko/cko^* mice to produce *Camk2α-cre^+/−^*; *Rapgef2^cko/cko^*. All drugs were administrated by intraperitoneal injection. D1 dopamine receptor agonist SKF81297 (2 or 5 mg/kg), psychostimulants D-amphetamine (10 mg/kg), and cocaine (30 mg/kg) were purchased from Sigma and dissolved in saline. The MEK inhibitor SL327 (60 mg/kg; Tocris) was dissolved in DMSO and then diluted twice in sterile water.

### Immunohistochemistry

Mice were perfused with ice-cold saline supplemented with 5 mM EGTA followed by 4% paraformaldehyde. After being postfixed with the same solution overnight, brains were sectioned by Vibratome at a 30- to 35-μm thickness. Free-floating sections were washed in TBS containing 0.5% Triton X-100 (TBST; three washes, 15 min), incubated at room temperature in blocking solution (10% normal goat serum in TBST; 1 h), and then incubated in primary antibody diluted in blocking solution overnight at 4°C. The following day sections were washed in TBST (three washes, 15 min), incubated in the dark in Alexa Fluor 555-conjugated goat anti-rabbit-IgG or Alexa Fluor 488-conjugated goat anti-mouse IgG (1:300; Life Technologies) for 2 h following primary antibody incubation. Sections were mounted in Vectashield (Vector Laboratories). The primary antibodies used were rabbit anti-pERK (1:1500, Cell Signaling), rabbit anti-pCREB (Ser133; 87G3; 1:1000, Cell Signaling), mouse anti-GAD67 (1:2500, Millipore), mouse anti-GFAP (clone 131-17719, 1:500, Molecular Probe), mouse anti-tyrosine hydroxylase (TH; 1:1000, Immunostar), mouse anti-MAP2a, b (clone AP20, 1:200, Lab Vision Corporation), and custom-made rabbit anti-Rapgef2 (NNLE-2). To test the specificity of Rapgef2 signals, Rapgef2 (NNLE-2) antibody was absorbed with Rapgef2 antigen peptide (keyhole limpet hemocyanin conjugated to LPADFTKLHLTDSLH with a cysteine linker) at 25 μM for 30 min at room temperature with end-over-end rotation, then subjected to immunohistochemistry with 1:2000. Confocal images were obtained on a Zeiss LSM 510. Images were converted to 16-bit greyscale and the number of pERK-positive cells in a 318 × 318 µm area in the dentate gyrus (DG) granule cell layer, basolateral amygdala (BLA), or the nucleus accumbens (NAc) was quantitated using the NIH ImageJ software. Images were thresholded to highlight positive neurons and subjected to Analyze Particles by setting “size (pixel ^2)” as 50-infinity.

### AAV-mediated Rapgef2 ablation in mouse NAc

Modified AAV vectors were obtained from Penn Vector Core. *Rapgef2^cko/cko^* mice were unilaterally injected with *AAV9.hSynap.HI.eGFP-Cre.WPRE.SV40*, encoding eGFP-fused cre recombinase under the control of the human synapsin promoter, to knock-out Rapgef2 expression in NAc on one side of the brain. *AAV9.hSynap.eGFP.WPRE.SV40*, encoding eGFP without Cre, also under the control of the synapsin promoter, was used as a control. Surgeries and viral injection were conducted according to the NIH-ARAC Guidelines for Survival Rodent Surgery. Briefly, animals were anesthetized, shaved, and mounted into the stereotaxic apparatus. A small midline incision was then made and the pericranial tissue was teased away from the skull with an ethanol-soaked swab to enable identification of the bregma and lambda areas. Using a small hand-held drill, a very small hole was made in the skull according to the calculated coordinates for NAc (from bregma, anterio-posterior 1.3 mm, medial-lateral 0.7 mm, dorsal-ventral 4.5 mm). A Hamilton syringe (preloaded with viral particles) was then slowly lowered, penetrating the dura to the determined depth. A volume of 0.5 μl of the concentrated virus (∼1 × 10^9^ infectious particle per microliter) was then slowly (∼0.1 μl/min) injected into the brain. A 2- to 3-min wait was performed before the needle was very slowly retracted from the brain to prevent backflow of the viral vector. The animals were allowed to recover and assessed for Rapgef2 ablation in the NAc four weeks after viral injection.

## Results

We previously reported that intracellular cAMP causes neurite extension (neuritogenesis) in NS-1 cells; that this requires expression of the protein product of the *Rapgef2* gene, NCS-Rapgef2; and that the human ortholog of this variant of Rapgef2 causes gain-of-function for ERK activation by cAMP in HEK293 cells (Hamada et al., 2012; Emery et al., 2013). We wished to determine if the acquisition of cAMP-dependent ERK activation through expression of NCS-Rapgef2 is a feature of cAMP→ERK signaling *in vivo* as well as in cell lines, and whether or not NCS-Rapgef2 expression is a specific feature of neuronal/endocrine cells and tissues in the adult mammal.

### Two classes of transcripts are emitted from the Rapgef2 gene, and one is neuroendocrine specific. Only transcript cluster 1’-containing tissues express NCS-Rapgef2 protein in the adult

We first wished to define clearly how various *Rapgef2* transcripts arise and whether differential transcription from this gene locus could contribute to distinct Rapgef2 translation products, potentially explaining the protean manifestation of cAMP-dependent and cAMP-independent, neuronally specific and nonspecific, Rapgef2/CNrasGEF/nRap-GEP/PDZ-GEF/recombinant Rapgef2 proteins reported in the literature to date.

**Figure 1.**
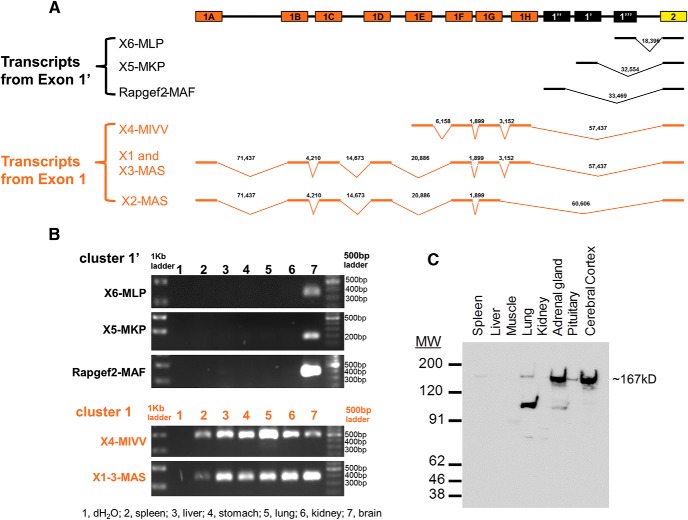
Two classes of transcripts from the Rapgef2 gene in mouse, one neuronal/endocrine and one non-neuronal/endocrine. ***A***, Schematic of mouse *Rapgef2* gene exons 1 and 2 and predicted transcripts (refer to https://www.ncbi.nlm.nih.gov/assembly/GCF_000001635.24/). Mouse *Rapgef2* gene exons are represented as boxes on top line. The lower schematics illustrate the alternative splicing of exons 1, producing different transcripts. Heavy lines represent the exons contained in each transcript and are connected by chevrons indicating alternative splicing among various exons 1. The numbers above each chevron indicate the base pair distance in the mouse genome between exons joined by splicing. The predicted transcripts are classified into two clusters: exon 1’ is denoted in black and exon 1 in orange. Note that all transcripts shown contain in common exons 2 and following exons (data not shown). Note exon 2 begins with the amino acid sequence LPAD (see text). Schematic of human *Rapgef2* gene exons 1 and 2 and predicted transcripts are shown in Extended Data Fig. 1-1*A*. ***B***, Differential expression of exons 1 and 1’ in mouse tissues by RT-PCR. Sense primers are specific to each mRNA transcript including X4-MIVV, X5-MKP, X6-MLP, and Rapgef2-MAF and a group of highly similar transcripts X1-3MAS. Antisense primers span the boundaries between exon 2 and exon 3. Sequences for primers are listed in Extended Data Fig. 1-2. The specific PCR products with the size corresponding to Rapgef2-MAF, X5-MKP, and X6-MLP, respectively, are detected only in brain (lane 7) but not in spleen, liver, stomach, lung, and kidney (lanes 2–6). The PCR products corresponding to X4-MIVV and X1-3-MAS are present in all tissue tested (lanes 2–7). Differential expression of exons 1 and 1’ in human cell lines are shown in Extended Data Fig. 1-1*B*. ***C***, Western blots using protein lysates from peripheral tissues and brain. A total of 30 µg of total proteins from brain and peripheral tissues were separated on gel and stained with rabbit polyclonal antibodies (NNLE-2) against the N terminus of Rapgef2. The full-length ∼167-kDa Rapgef2 protein (NCS-Rapgef2) is unambiguously detected only in neuronal and neuroendocrine tissues (e.g., brain, pituitary, adrenal gland). Blots were stained with Ponceau S to confirm qualitatively that the same amount of protein was present in each lane, as determined initially by loading standard protein concentrations (as determined by BCA assay) to each lane.

Bioinformatic analysis of all possible transcripts from the mouse and human *Rapgef2* genes reported in Genbank are shown in Figure 1*A* and Extended Data Fig. 1-1A. All *Rapgef2*-related transcripts reported, from all tissues of the mouse and including mouse embryo, contained the exon beginning LPAD in the *Rapgef2* ORF, which we have now termed “exon 2,” and continue to ORF exon 24. Variants of mouse *Rapgef2* contain multiple combinations of 11 upstream exons, including the 5’ most exon, 75 kb upstream of the next nearest downstream one, and previously not assigned in RefSeq to the *Rapgef2* gene locus. We have designated these exons as belonging to two transcript families, termed by us the exon 1, and the exon 1’ clusters, based on extensive amplicon analysis in mouse tissues (Fig. 1*B*). What distinguishes exon family 1 transcripts from exon 1’ transcripts are that the former are present in all tissues examined, whereas the latter are found only in neuronal tissues (Fig. 1*B*). The human *Rapgef2* gene locus is similarly arranged (Extended Data Fig. 1-1A), except that there is a single identified first exon in the 1’ transcript family, similarly expressed in neuronal/endocrine cells.

10.1523/ENEURO.0248-17.2017.f1-1 Figure 1-1 Two classes of transcripts are alternatively used from the *Rapgef2* gene in human. *A*, Schematic of human *Rapgef2* gene exons 1 and 2 and predicted transcripts (refer to https://www.ncbi.nlm.nih.gov/assembly/GCF_000001405.33/). Human *Rapgef2* gene has similar genomic structure and alternative splicing pattern as mouse. The exons are represented as boxes on the top genomic diagram and by heavy lines in each transcript. Alternative splicing products are depicted as chevrons connecting different exons 1. The base pair distance between two joined exons in the genome is indicated above the chevrons. The putative transcripts are classified into two clusters: exon 1’ denoted with black color and exon 1 with an orange color. The translational start codon ATG is marked for each transcript with an arrow. *B*, Differential expression of exons 1 and 1’ in human cell lines by RT-PCR. Exon-specific sense primers for mRNA transcript X9 MIVV and highly similar transcripts X1-7MAS, X13-14 are used. Antisense primers span the boundaries between exon 2 and exon 3. The specific PCR product of the size corresponding to X13-14 is absent in HEK293 cells but is detected in neuroblastoma SY5Y cells. The PCR products corresponding to X1-7 and X9 are present in both cell lines. Download Figure 1-1, TIF file.

**Figure 2.**
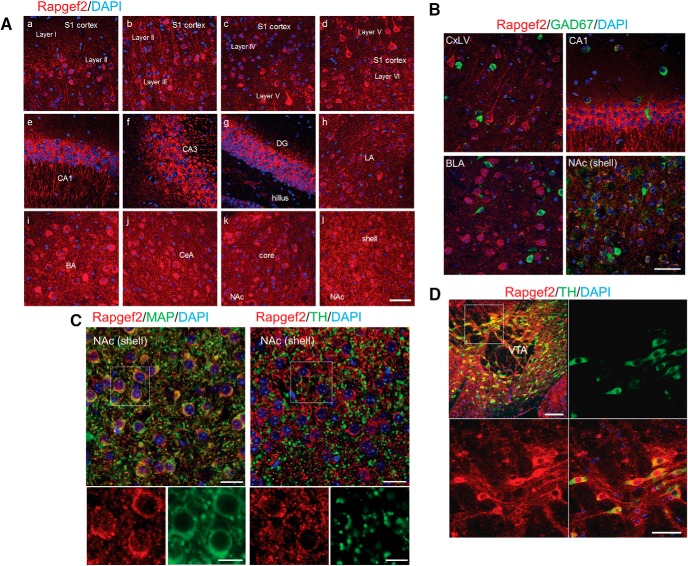
NCS-Rapgef2 protein distribution in brain. ***A***, Expression of NCS-Rapgef2 protein in C57BL6/N brain. Immunohistochemistry using Rapgef2 N-terminal-specific antibody NNLE-2 (see Extended Data Fig. 2-1A for the characterization of this antibody) indicated that Rapgef2 protein (red) is highly expressed in the neurons of cortex, hippocampus, striatum, amygdala, and other brain regions. With counterstaining by DAPI (blue), Rapgef2 protein (red) was visualized in cytoplasm, associated with cell membrane. Scale bar: 50 μm. More expression results can be found in Extended DataFig. 2-1. ***B***, NCS-Rapgef2 is predominantly expressed in the excitatory neurons of corticolimbic areas and the MSNs of striatum. Brain sections are double-stained with Rapgef2 (red) and GAD67 (green) antibodies. CxLV, layer V of S1 cortex. Scale bar: 50 μm. ***C***, In the NAc, NCS-Rapgef2 is expressed in the soma and dendrites (indicated by MAP2 staining) of the postsynaptic MSNs of dopaminergic terminals which are indicated by TH staining. Scale bar: 20 μm (upper panel) and 10 μm (lower panel). ***D***, NCS- Rapgef2 protein is expressed in TH-positive dopaminergic neurons in VTA. Scale bar: 100 μm (upper panel) and 50 μm (lower panel).

10.1523/ENEURO.0248-17.2017.f1-2 Figure 1-2 Primers used to detect mouse (m) and human (h) Rapgef2 mRNA isoforms. Download Figure 1-2, TIF file.

Commercially-available antibodies against Rapgef2 produced bands besides the native 167-kDa full-length protein that could not be reliably distinguished as either breakdown products of Rapgef2 itself, or nonspecific signals (data not shown). In addition, cross-reactivity with the highly homologous protein Rapgef6, with some antibodies, could only be ruled out on Western blotting due to the larger size of the latter. We therefore generated a set of rabbit polyclonal antibodies against the N terminus of Rapgef2 (the “LPAD” epitope), and characterized them for Western blotting (Fig. 1*A*) and immunohistochemistry (Extended Data Fig. 2-1A). One of these antibodies, NNLE-2, against the N-terminal domain of Rapgef2, was used to reveal that the full-length 167-kDa mouse Rapgef2 protein is detected by Western blotting only in brain and endocrine tissues (pituitary and adrenal), and not in spleen (except in trace and variable amounts), liver, skeletal muscle, or kidney (Fig. 1*C*). Thus, Rapgef2 protein expression corresponds to expression of exon 1’-containing transcripts, with no protein expression in tissues expressing only exon 1-containing transcripts.

10.1523/ENEURO.0248-17.2017.f2-1 Figure 2-1 NCS-Rapgef2 protein expression in mouse brain. *A*, Characterization of Rapgef2 antibody NNLE2 by immunohistochemistry. Upper panel, Mouse cortex stained for NCS-Rapgef2 with NNLE-2 antibody in the absence (left) or presence (right) of the blocking peptide. Lower panel, NCS-Rapgef2 staining with NNLE-2 antibody four weeks after the NAc on one side of the brain of floxed Rapgef2 mouse Rapgef2^cko/cko^ was injected with AAV9.hSyn.HI.GFP-Cre virus. Scale bar: 20 µm. *B*, Western blottings using tissues from different areas of CNS showed high level of NCS-Rapgef2 protein expression in hippocampus, cortex, striatum, etc. ***C***, Expression of NCS-Rapgef2 protein in C57BL6N brain. Immunohistochemistry using Rapgef2 N-terminal-specific antibody NNLE-2 indicated that Rapgef2 protein (red) is highly expressed in the neurons of cortex, hippocampus, striatum, amygdala, and other brain regions. ***D***, NCS-Rapgef2 protein is predominantly expressed in the excitatory neurons of corticolimbic brain areas indicated by none overlapping staining of Rapgef2 and inhibitory neuron marker GAD67 in these areas. NCS-Rapgef2 protein is highly expressed in medial spiny neurons in striatum indicated by localization of Rapgef2 in the neurons with weak GAD67 staining in striatum. Immunostaining of NCS-Rapgef2 is not localized in astrocytes as indicated by GFAP staining. Download Figure 2-1, TIF file.

### Cellular localization of NCS-Rapgef2 in mouse brain

Immunohistochemistry with brain sections from C67BL6/N mice shows neuronal staining of NCS-Rapgef2 with the NNLE-2 antibody, but not in the presence of the blocking peptide that was used to generate the antibody (Extended Data Fig. 2-1A, upper panels). In the NAc of a Rapgef2^cko/cko^ mouse generated with loxP sites flanking exon 4 of the Rapgef2 gene locus (see the later Fig. 5*A*), injection of an AAV vector, *AAV9.hSynap.HI.eGFP-Cre.WPRE.SV40*, results in almost complete loss of Rapgef2 staining, compared to the uninjected side of the ventral striatum of the same mouse (Extended Data Fig. 2-1A, lower panels).

Both Western blotting (Extended Data Fig. 2-1B) and immunohistochemistry (Fig. 2*A*; Extended Data Fig. 2-1C,*D*) results indicate high expression of NCS-Rapgef2 protein in CNS, especially in the neurons of cortex, hippocampus, amygdala, striatum, and cerebellum. NCS-Rapgef2 immuno-signals were predominantly visualized in the cytoplasm, and excluded from the nucleus, of corticolimbic excitatory neurons (Fig. 2*A*,*B*), striatal medial spiny neurons (Fig. 2*B*,*C*), and TH-positive dopaminergic neurons in ventral tegmental area (VTA; Fig. 2*D*), and enriched on dendrites (Fig. 2*A–D*), consistent with a postsynaptic role for NCS-Rapgef2 signaling in the brain.

### NCS-Rapgef2 gain-of-function for ERK activation and dependence on the CNBD

**Figure 3.**
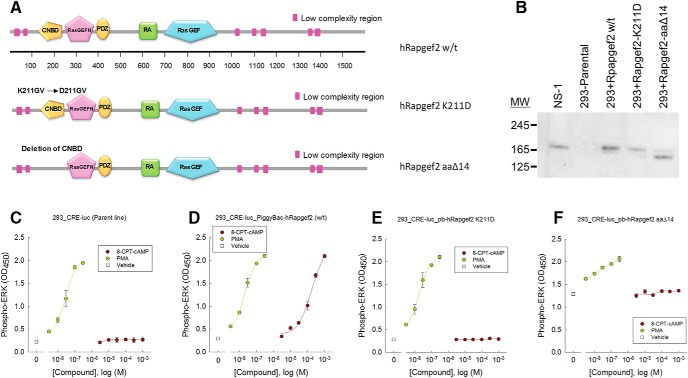
Rapgef2 structural analysis by ERK signaling gain-of-function in HEK293 cells. ***A***, Various proposed functional cassettes of NCS-Rapgef2. CNBD: cAMP/cGMP binding doman (Pham et al., 2000); RasGEFN: N-terminal motif, common to proteins containing RasGEF (Cdc25-like) domains, reported to play a structural role (Boriack-Sjodin et al., 1998); PDZ: the PDZ-binding domain possibly allowing Rapgef2 interaction with certain GPCRs (Pak et al., 2002); RA: Ras/Rap-associating domain is a conserved domain that stimulates GDP dissociation from small GTPases (Hofer et al., 1994); RasGEF: GEF activity domain, GEF activity has been reported for both Ras and Rap (Pham et al., 2000); or to be specific for Rap1 and Rap2 (de Rooij et al., 1999). Human Rapgef2 K211D made by mutating the lysine codon to an aspartate codon in the human KGV motif of the Rapgef2 CNBD which abolishes binding to cAMP. Human Rapgef2 aaΔ14 was made by deletion of 14 residues within the CNBD. ***B***, Western blotting profile of Rapgef2 protein abundance in assessed cell lines, as a reference, neuroendocrine cell lysates (NS-1, lane 1) were included. Lane 2, parental HEK293 cell line; lane 3, HEK293 cell line after stable introduction of wild-type human Rapgef2; lane 4, HEK293 cell line expressing K211D Rapgef2 mutant; lane 5, HEK293 cells expressing aaΔ14 Rapgef2 mutant. ***C***, cAMP->ERK signaling is absent in HEK293 cells. ***D***, Constitution of cAMP->ERK signaling is absent in HEK293 cells expressing wild-type human NCS-Rapgef2. ***E***, Loss of Rapgef2 function in cells expressing the K211D mutant. ***F***, Constitutive ERK activation in HEK293 cells expressing hRapgef2 aaΔ14.

Having established that a neuronally-specific transcript encodes a full-length Rapgef2 protein, we sought to characterize the signaling function of this protein in a non-neuronal cell using a gain-of-function assay, and to determine the structural requirements for its function. We constructed a series of hRapgef2 mutations (cluster exon 1’; Extended Data Fig. 1-1A) expressed in PiggyBac vectors (Fig. 3*A*, detailed descriptions are in Materials and Methods). For gain-of-function experiments using these constructs, we chose the non-neuronal cell line, HEK293, that expresses the cluster 1 exon-expressing *Rapgef2* mRNA (Extended Data Fig. 1-1B), but no Rapgef2 protein (Fig. 3*B*), also see reference (Emery et al., 2013). By Western blotting, we confirmed that HEK293-based cell lines with stably-integrated Rapgef2 ORFs initiated from exon 1’ expressed Rapgef2 protein at a similar abundance to NS-1 cells (Fig. 3*B*). As shown in Figure 3*C*,*D*, stable introduction of wild-type human NCS-Rapgef2 into the HEK293-CRE-luc parent line caused gain of cAMP-dependent ERK signaling in this cell background. Note that PMA treatment, a control for cAMP-independent ERK activation in this cell line, results in equivalent elevation of phospho-ERK across all of the cell lines tested. To determine if the predicted cyclic nucleotide monophosphate binding cassette (CNBD; Fig. 3*A*) is necessary for function, an NCS-Rapgef2 with a point mutation in the CNBD (K211D) known to abrogate cAMP binding in other CNBD cassettes (Pham et al., 2000) was constructed. NCS-Rapgef2-K211D introduced into HEK293 cells failed to support cAMP-ERK coupling (Fig. 3*E*). A deletion of the CNBD region (aaΔ14) was also performed. Expression of the aaΔ14 Rapgef2 deletion mutant in HEK293 cells resulted in high constitutive ERK phosphorylation (Fig. 3*F*). Thus, the CNBD is necessary for cAMP to exert control over ERK signaling through the intermediacy of NCS-Rapgef2, and this region appears to function as an auto-inhibitory domain for the GDP-releasing enzymatic activity of NCS-Rapgef2, with inhibition relieved by binding of cAMP.

### NCS-Rapgef2 couples D1-receptor-dependent cAMP elevation to ERK activation in NS-1 cells

We have previously shown that the Gs-coupled GPCR PAC1, activated by pituitary adenylate cyclase-activating polypeptide (PACAP), activates ERK through Rapgef2 to cause neuritogenesis in NS-1 cells (Emery et al., 2013). However, the distribution pattern of NCS-Rapgef2 in the central nervous system is not sufficiently restricted as to suggest a private role for NCS-Rapgef2 in PACAPergic neurotransmission in brain. We therefore wondered if other Gs-coupled GPCRs might employ NCS-Rapgef2 signaling, and began to explore this possibility. Since dopamine neurotransmission is critical in corticolimbic and striatal regions where Rapgef2 expression in mouse brain is high, we examined whether or not cAMP elevation following D1 receptor stimulation would result in Rapgef2-coupled ERK activation and neuritogenesis in NS-1 cells.

**Figure 4.**
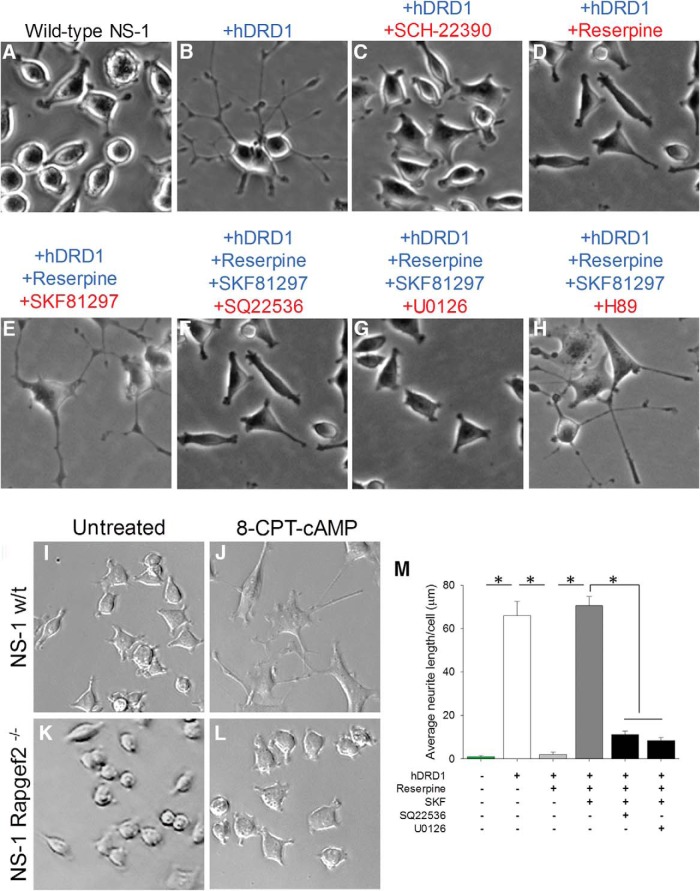
NCS-Rapgef2 couples D1 receptor-dependent cAMP elevation to ERK activation in NS-1 cells. NS-1 cells (***A***) were transduced with the lentiviral pL304-hDRD1 (human D1 dopamine receptor) vector and a stable cell line was generated by antibiotic selection. DRD1-expressing cells extended neurites (***B***). Neuritogenesis was blocked by the addition of D1 dopamine receptor antagonist 10 µM SCH-22390 (***C***) or 10 µM reserpine (***D***). Neuritogenesis was restored in cells grown in media containing 10 µM reserpine following treatment with (***E***) 10 µM SKF81297 for 48 h. The neuritogenic effect of SKF81297 was inhibited by cotreatment with either (***F***) 1 mM SQ22536, or (***G***) 10 µM U0126, but not by 30 µm PKA inhibitor H89 (***H***). Seventy-two hours of exposure to 8-CPT-cAMP (300 μM) promotes neuritogenesis in wild-type NS-1 cells (***I***, ***J***), but not in NS-1 cells in which both alleles of the *Rapgef2* gene were deleted (***K***, ***L***). Images in ***I–L*** are representative of experiments repeated eight times. ***M***, Quantification of neurite outgrowth assays in NS-1 cells transduced with D1 dopamine receptor or treated with different drugs. Data shown in the histograms are mean ± SEM (*n* = 12 per group). *F*_(5,66)_ = 95.66, *p < 0.001, one-way ANOVA followed by *post hoc* Bonferroni *t* test.

Human D1 dopamine receptor-expressing NS-1 stable cell lines were generated using a lentiviral vector encoding an hDRD1a receptor (GeneCopoeia) and the blasticidin S resistance gene *bsr*. DRD1a-positive NS-1 cells spontaneously extend neurites, presumably driven by the presence in the medium of dopamine generated by the NS-1 cells themselves (Fig. 4*A*,*B*). To grow and passage this cell line, media were supplemented with 10 μM haloperidol, which blocked neurite elongation. For experiments, cells were plated in media with 10 μM haloperidol, which was washed off the next day and replaced with fresh media (Fig. 4*B*). Spontaneous neurite elongation of hDRD1-expressing NS-1 cells was inhibited in medium containing the D1R antagonist SCH-22390 (Fig. 4*C*) or in medium containing 10 μM reserpine to deplete endogenous dopamine (Fig. 4*D*). In dopamine-depleted (reserpine-treated) cells, neuritogenesis was stimulated by treatment with 10 μM of the D1 agonist SKF81297 (Fig. 4*E*), and the neuritogenic effect of SKF81297 was inhibited by treatment with the adenylate cyclase and Rapgef2 inhibitor SQ22536 (1 mM; Fig. 4*F*) or 10 μM of the MEK inhibitor U0126 (Fig. 4*G*), but not with the PKA inhibitor H89 (30 μM; Fig. 4*H*). These results demonstrated that NCS-Rapgef2 couples D1 receptor-dependent cAMP elevation to ERK activation which leads to neuritogenesis in NS-1 cells.

To confirm that Rapgef2 is necessary for cAMP-dependent neuritogenesis, and is therefore a requisite component of the signaling pathway from cAMP to ERK, we used guide RNAs to target the *Rapgef2* gene locus, in an NS-1 line stably expressing Cas9 nuclease, to generate a cell line devoid of Rapgef2 protein expression. Using this approach clonal cell lines were generated and screened by PCR for biallelic editing of *Rapgef2*. In wild-type NS-1 cells, treatment with the cell-permeable cAMP analog 8-CPT-cAMP (300 μM) caused robust neurite outgrowth (Fig. 4*I*,*J*). By contrast, in cells devoid of Rapgef2 (Fig. 4*K*,*L*), 8-CPT-cAMP did not cause neurite outgrowth, indicating that Rapgef2 expression is indeed required for the cAMP- and ERK-dependent process of neuritogenesis (Emery and Eiden, 2012). Figure 4*M* represents quantification of the representative data shown in Figure 4*A*,*B*,*D-G*.

### Dopaminergic NCS-Rapgef2-dependent ERK activation in mouse hippocampus and amygdala

**Figure 5.**
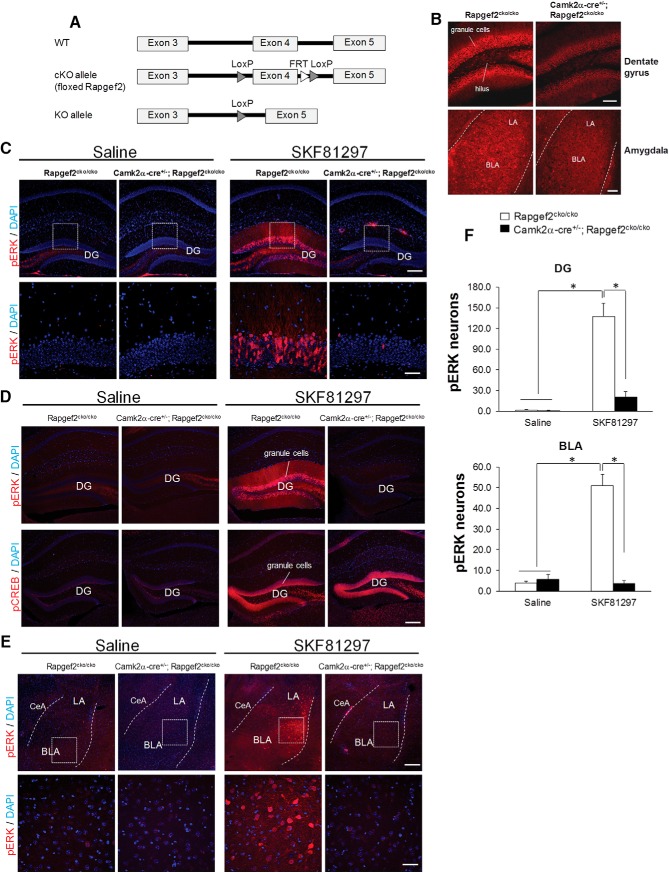
Dopaminergic NCS-Rapgef2-dependent ERK activation in mouse hippocampal DG and amygdala. ***A***, Characterization of Rapgef2 cKO allele by sequence blast of primers for genotyping and subcloning of the sequence between two loxp sites. The Rapgef2 conditional targeting area is within the exon 4 cAMP-binding domain. ***B***, Coronal sections from 20-week-old *Rapgef2^cko/cko^* and *Camk2α-cre^+/−^*; *Rapgef2^cko/cko^* mice were immunostained with Rapgef2 NNLE-2 antibody. NCS-Rapgef2 protein levels were largely ablated in hippocampal DG area (upper panel) and lateral and basolateral amygdala (lower panel). Scale bars: 100 µm. ***C–E***, Rapgef2-dependent ERK activation and phosphorylation in the hippocampal DG and BLA induced by D1 dopamine receptor agonist SKF 81297. *Rapgef2^cko/cko^* or *Camk2α-cre^+/−^*; *Rapgef2^cko/cko^* mice were treated with saline or SKF 81297 (5 mg/kg, i.p.). Thirty minutes after injection, animals were perfused, and the coronal sections were immunostained with phospho-ERK antibody. Phosphorylation of ERK (red signal) was significantly induced by SKF81297 in the neurons of dentate granule cell layer (***C***, ***D***) and BLA (***E***) in *Rapgef2^cko/cko^* mice, but not in *Camk2α-cre^+/−^*; *Rapgef2^cko/cko^* mice. However, phosphorylation of CREB in the hippocampal dentate granule cell layers induced by SKF81297 was not NCS-Rapgef2-dependent (***D***). In ***C***, ***E***, lower panels are pictures with higher magnification from the areas with white frame in the corresponding upper panels. In ***D***, lower panels are phospho-CREB staining using brain sections from the same animal used for phospho-ERK staining shown in upper panels. Scale bar: 200 µm (upper panel) and 50 µm (lower panel, ***C***, ***E***), 200 µm (***D***). ***F***, Quantification of phospho-ERK immunoreactive neurons in hippocampal DG (upper panel) and basolateral amygdala (lower panel) of *Rapgef2^cko/cko^* or *Camk2α-cre^+/−^*; *Rapgef2^cko/cko^* mice treated with saline or SKF81297 (5 mg/kg) and perfused 30 min later. A two-way ANOVA was run on a sample of 16 animals to examine the effect of genotype and drug treatment on phosphorylation of ERK. There was a significant genotype effect on phospho-ERK in DG (*F*_(1,12)_ = 33.14, *p* < 0.0001) and in BLA (*F*_(1,12)_ = 56.53, *p* < 0.0001). There was a significant interaction between the effects of genotype and drug treatment on phospho-ERK in DG (*F*_(1,12)_ = 32.03, *p* < 0.0001) and in BLA (*F*_(1,12)_ = 65.56, *p* < 0.0001) as well. Data shown in the histograms are mean ± SEM (*n* = 4 mice per group). *p < 0.001, two-way ANOVA followed by *post hoc* Tukey HSD test.

D1 dopamine receptor-dependent signaling via ERK activation is critical in modulating the balance of excitatory/inhibitory transmission, synaptic plasticity, and memory formation in hippocampus (Sweatt, 2004; Gangarossa et al., 2011; Bozzi and Borrelli, 2013; Werlen and Jones, 2015). ERK activation in amygdala is also critical for memory formation and drug addiction (Schafe et al., 2000; Lu et al., 2005; Radwanska et al., 2005; Luo et al., 2013). To examine whether Rapgef2 is involved in D1 receptor-mediated activation of ERK in hippocampus and amygdala, Rapgef2 was ablated postnatally in forebrain excitatory neurons by crossing *Rapgef2^cko/cko^* (Fig. 5*A*) with *Camk2α-cre* (*T29-1*) transgenic mice. Immunohistochemistry using Rapgef2 antibody indicated the loss of Rapgef2 expression in granule cells of hippocampal DG, excitatory neurons in lateral (LA) and basolateral amygdala (BLA) in adult mice (Fig. 5*B*). D1 agonist SKF81297 (5 mg/kg, i.p.) induced robust ERK phosphorylation in D1R-positive neurons in the DG granule cell layer, in LA, and in BLA of *Rapgef2^cko/cko^* mice (Fig. 5*C*–*F*). However, phospho-ERK induction by SKF81297 was significantly attenuated in the DG and amygdala of *Camk2α-cre^+/−^*; *Rapgef2^cko/cko^* mice (Fig. 5*C–F*). Interestingly, phospho-CREB induction in dentate granule cells after SKF81297 was not affected by NCS-Rapgef2 ablation (Fig. 5*D*). These results demonstrate the existence of the signal pathway of D1R→Rapgef2→pERK in hippocampal DG and amygdala.

### Dopaminergic NCS-Rapgef2-dependent ERK activation in mouse NAc

**Figure 6.**
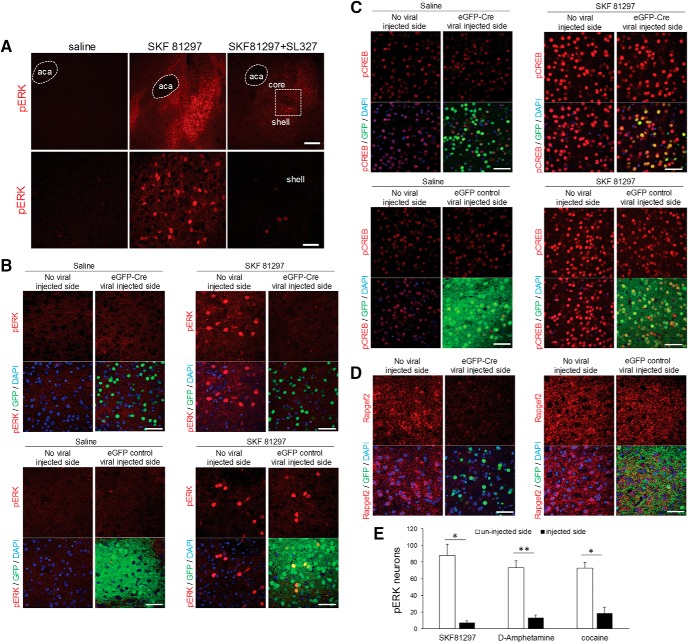
NCS-Rapgef2-dependent ERK activation in mouse NAc following D1 dopamine receptor agonist SKF 81297 and psychostimulants D-amphetamine or cocaine administration. ***A***, C57BL6N wild-type mice received either saline or D1 dopamine receptor agonist SKF 81297 (2 mg/kg, i.p.). Phosphorylation of ERK in the NAc, especially in the shell, was robustly induced 15 min after D1 agonist treatment. A systemic injection of MEK inhibitor SL327 (60 mg/kg, i.p.) 60 min before treatment with SKF 81297 significantly reduced phosphorylation of ERK in NAc. Lower panels are pictures with higher magnification from the shell areas in upper panels (indicated by the white frame). Scale bar: 200 µm (upper panel) and 50 μm (lower panel). ***B–D***, AAV viral vector-directed ablation of *Rapgef2* expression in the NAc impaired ERK activation induced by D1 dopamine receptor agonist SKF 81297. *Rapgef2^cko/cko^* mice were unilaterally injected with *AAV9.hSynap.HI.eGFP-Cre.WPRE.SV40* or control viral vector *AAV9.hSynap.eGFP*, which encodes eGFP-fused cre recombinase or eGFP alone under the control of human synapsin promoter. Four weeks later, animals were treated with saline or SKF81297 (2 mg/kg, i.p.) and perfused 15 min later. Phosphorylation of ERK (***B***) induced by SKF81297 in the NAc was absent on the side of the brain with cre viral injection (shown by nuclear eGFP signal) (upper panels), but clearly present in the NAc injected with control eGFP viral vector (lower panels). Phospho-CREB (***C***) induced by SKF81297 in the NAc on either the side of the brain with cre injection (upper panels) or on the side of the brain receiving control viral vector (lower panels) administration. Loss of NCS-Rapgef2 protein expression (***D***) was observed in the NAc of *Rapgef2^cko/cko^* mice four weeks following unilateral injection of *AAV9.hSynap.HI.eGFP-Cre.WPRE.SV40* (left panel), but not control viral vector (right panel). Scale bar: 50 μm. ***E***, *Rapgef2^cko/cko^* mice were unilaterally injected with *AAV9.hSynap.HI.eGFP-Cre.WPRE.SV40* into the NAc. Neurons showing phospho-ERK after SKF 81297(2 mg/kg, i.p.), D-amphetamine (10 mg/kg, i.p.), or cocaine (30 mg/kg, i.p.) were counted using the NIH ImageJ software. Cell counts represent the average obtained from 3three to five animals per treatment group measured in a 318 × 318 μm area in the NAc. Student’s *t* test showed significant reduction of ERK phosphorylation on viral vector injected side for all drug treatment groups (*p < 0.01, **p < 0.001) where Rapgef2 expression was ablated, compared to uninjected side, of the NAc.

D1 agonists and psychoactive stimulants, such as D-amphetamine and cocaine, robustly induce the rapid phosphorylation and activation of ERK1/2 in D1R- expressing medium spiny neurons (MSNs) of the NAc (Valjent et al., 2000; Bertran-Gonzalez et al., 2008; Gerfen et al., 2008; Pascoli et al., 2014). ERK activation appears critical for the sensitization of D1 responses that contributes to addiction. We first confirmed that phosphorylation of ERK in the NAc was robustly induced 15 min after D1 agonist SKF 81297 (2 mg/kg, i.p.) treatment. A systemic injection of MEK inhibitor SL327 (60 mg/kg, i.p.) 60 min before treatment with SKF81297 significantly reduced phosphorylation of ERK in the NAc (Fig. 6*A*). To determine if the cAMP/NCS-Rapgef2 signaling pathway mediates D1-dependent activation of ERK in the NAc, an AAV vector expressing Cre recombinase fused with eGFP under the control of the synapsin promoter (*AAV9.hSynap.HI.eGFP-Cre.WPRE.SV40*), or control viral vector (*AAV9.hSynap.eGFP.WPRE.SV40*), which encodes eGFP only under hSyn promoter, were unilaterally injected into the NAc of *Rapgef2^cko/cko^* mice. Four weeks later, these mice were treated with saline or the selective D1 agonist SKF 81297 (2 mg/kg, i.p.). ERK activation 15 min following D1 agonist treatment was significantly reduced on the eGFP-Cre virus-injected side (shown by nuclear eGFP signal; Fig. 6*B*, upper panels), but not on the eGFP control viral vector-injected side (Fig. 6*B*, lower panels). SKF 81297-induced CREB phosphorylation in NAc, on the other hand, was not affected by knock-out of Rapgef2 expression (Fig. 6*C*). Finally, NCS-Rapgef2 gene deletion in the experiments described above was confirmed, by loss of staining for the NCS-Rapgef2 protein in the NAc by cre-loxP mediated gene knock-out with eGFP-Cre viral injection, but not eGFP control viral injection (Fig. 6*D*). Similar effects were observed when mice were treated with psychostimulants D-amphetamine (10 mg/kg, i.p.) or cocaine (30 mg/kg, i.p.) after *AAV-cre* virus-directed Rapgef2 ablation in NAc (Fig. 6*E*).

These data demonstrate that D1 agonist or psychostimulants-induced pERK in the NAc is Rapgef2-dependent. We propose that the cAMP-dependent pathway for ERK activation initiated at the D1 receptor in the brain most likely proceeds via activation by NCS-Rapgef2 of the Rap1B→B-Raf→MEK1/2 signaling cassette, as shown in NS-1 cells (this report; Emery et al., 2013; Emery et al., 2017a; Emery et al., 2017b). However, it may yet be that another Rap (e.g., Rap1A), another MAP kinase kinase kinase besides B-Raf (e.g., C-Raf), or a specific MEK (e.g., either MEK1 or MEK2) isoform may participate in the pathway from D1 receptor activation through NCS-Ragpef2 to ERK activation in D1 dopaminoceptive neurons *in vivo*. We note further that this signaling pathway is apparently supported equally efficaciously via D1 receptor activation through Gs (NS-1 cells; hippocampus) and Golf (NAc).

## Discussion

We show here that the guanine nucleotide exchange factor NCS-Rapgef2 is encoded by a neuron-specific transcript from the *Rapgef2* gene locus, and functions as an obligate cAMP sensor for dopamine-dependent ERK activation in hippocampus, amygdala, and ventral striatum. This work represents an extension of our finding that in cultured cells, NCS-Ragpef2 links cAMP elevation to ERK activation, to the demonstration that a specific catecholamine receptor, the dopamine D1 receptor, uses this specific signaling pathway for ERK activation, and that it is relevant to central nervous system ERK activation *in vivo*.

The *Rapgef2* gene locus and its putative protein products have been studied in a variety of cellular contexts over the past few decades. Rapgef2 was initially identified as a hypothetical human protein of ∼170 kDa with an N-terminal sequence of MKPLAIPANHGVMGQQEKHS and called KIAA0313 by the Kazusa DNA Research Institute in the mid-1990s (Wain et al., 2002). The first reported protein product of the *Rapgef2* gene locus was called nRap GEP (neural Rap guanine nucleotide exchange protein) based on its exclusive expression in neurons in the rodent brain, and its ability to catalyze GDP/GTP exchange on the small GTPase Rap, *in vitro* (Ohtsuka et al., 1999). A homolog of KIAA0313/nRap-GEP was discovered in nematode by Liao and colleagues in the same year, and was called RA-GEF-1. In this report, a recombinant preparation of the human protein ortholog was found to catalyze constitutive GDP release from Rap1A (Liao et al., 1999). Subsequent to the characterization of nRap GEP/RA-GEF-1, de Rooij et al. (1999) reported that a human cDNA encoding the same putative protein encoded a 200-kDa protein whose mRNA was ubiquitously expressed in human tissues, and which possessed Rap1/2 GEF activity when expressed as a recombinant protein *in vitro* and *in cellula* that was not enhanced by addition of cAMP or cGMP (subsequently confirmed by Rebhun et al., 2000), and called the protein PDZ-GEF1. Finally, the Rotin laboratory identified a mouse 130 residue protein 95% similar to the corresponding region of KIAA0313 and generated an ∼180-kDa recombinant holoprotein corresponding to this transcript, which was found to stimulate GDP release from Ras in a cGMP- and cAMP-dependent manner, and from Rap1 in a cAMP/cGMP-independent fashion. This protein was named CNrasGEF, for cyclic nucleotide ras GEF (Pham et al., 2000; Amsen et al., 2006). Hisata et al. (2007), subsequently proposed that the Rapgef2 protein has a functional role to enhance neurite outgrowth in PC12 cells initiated by neurotrophin signaling, through sustained linkage of ERK and Rap1 in late endosomes initiated at the TrkA receptor.

Despite the multiple, and often disparate biochemical characteristics of Rapgef2 (also known as KIAA0313/nRAP-GEP/PDZ-GEF1/CNrasGEF) determined *in vitro* and *in cellula* using recombinant versions of the protein, work reported from knock-out organisms has indicated a major physiologic role for this protein. The *Drosophila* ortholog, called Rap-GEF, has been shown to control stem cell niche anchoring in testis through regulation of cell adhesion (Wang et al., 2006). Deletion of *Rapgef2* in the mouse is embryonic (E7.5) lethal, due to failure to form either a yolk sac primary vascular plexus, or major blood vessels (Wei et al., 2007). Mice deficient in *Rapgef2* expression only in forebrain survive through birth, but have severe defects in brain structure and increased susceptibility to pilocarpine-induced seizure, suggesting a profound defect in neural migration during development (Bilasy et al., 2009). The midline structural defects (paucity of both callosal and hippocampal commisural fibers) seen in *Rapgef2*-deficient mice during development was traced to absence of glia, and the glial sling, at the cortical midline (Bilasy et al., 2011). Studies using a conditional knock-out strategy for the *Rapgef2* gene locus in mice have demonstrated that Rapgef2 is essential for embryonic hematopoiesis; yet is dispensable for hematopoiesis and endothelial cell function in adult mice (Satyanarayana et al., 2010).

In contrast to the pleiotropic roles that Rapgef2 fulfills during development, its potential functions in postdevelopmental neurons are somewhat more discrete. In hippocampal neurons, *ex vivo*, Rapgef2 is phosphorylated by Plk2, a postsynaptic kinase, allowing an association with SPAR, a postsynaptic regulator of Rap activity (Lee et al., 2011). Interactions within the protein complex of Rapgef2, PDZ-Zip70, and SPAR have been shown to be required for Rap2 activation in hippocampal neurons, which controls dendritic spine formation at glutamatergic postsynapses (Ryu et al., 2008; Mayanagi et al., 2015).

NCS-Rapgef2 protein is expressed only in neuronal and endocrine cells in adult mice, and we reveal here a likely mechanism for this. It is that *Rapgef2* gene transcription from the exons we have designated as exons 1 leads to transcripts generating unstable protein, or are not translated, and *Rapgef2* gene transcription from the exons we have designated as exons 1’, found only in neurons and endocrine cells, produces mRNA from which a stable Rapgef2 protein (NCS-Rapgef2) is translated. The mechanisms through which non-neuronal Rapgef2 (NN-Rapgef2) mRNA remains untranslated, or is translated and degraded, are unknown. Possibilities include (1) exon 1-encoded tagging of the non-neuronal Rapgef2 protein for ubiquitylation and degradation, and/or (2) exon 1-encoded translational blockade of NN-Rapgef2 mRNA. It is noteworthy that failure of degradation of Rapgef2 by the proteasome following ubiquitylation in epithelial cells results in sustained Rap1 activation and inhibition of hepatocyte growth factor (HGF)-induced cell migration (Magliozzi et al., 2013). In our view, these data support a role for degradation of NN-Rapgef2 protein in non-neuronal cells, after development, as a mechanism for assignment of basal Rapgef2 function uniquely to neuronal and endocrine signaling (i.e., to NCS-Rapgef2) in adult mammals, but with preservation of a role for NN-Rapgef2 in cellular adhesion and other functions, under paraphysiological or pathophysiological conditions, in adult non-neuronal tissues and cells (de Rooij et al., 1999; Amsen et al., 2006). In addition, non-cAMP-dependent functions of NN-Rapgef2 clearly evident during development (e.g., for stem cell migration, angiogenesis, early brain development, wound healing) may be preserved, in some cases, in adult non-neuronal tissues (vide supra; Bilasy et al., 2009; Satyanarayana et al., 2010;Pannekoek et al., 2011).

We postulate further that the full activation of NCS-Rapgef2 by cAMP in intact cells, compared to either lack of effect of cAMP or activation of Ras instead of Rap by cAMP measured *in vitro* (de Rooij et al., 1999; Pham et al., 2000) may be due to the need for additional protein(s), perhaps recruited via the CNBD-adjacent PDZ domain, to allow correct cAMP-dependent rotation of the CNBD away from the Rap1-interacting domain. In any event, the data presented here offer unequivocal evidence that NCS-Rapgef2 acts in cellula as a cAMP sensor dependent on a critical conserved amino acid within the CNBD for ERK activation. Our data also offer a plausible mechanism for this cAMP dependence, i.e., CNBD auto-inhibition of GEF activation that is relieved by cAMP, similarly to the mechanism proposed for the action of cAMP in activation of the Rapgefs Epac1 and Epac2 (Rehmann et al., 2006).

How is the neuron-specific expression of NCS-Rapgef2 relevant to Gs-GPCR-coupled signaling in the central nervous system? We have previously demonstrated PACAP→PAC1→cAMP→NCS-Rapgef2→ERK in neuroendocrine cells in culture, and now show here that this pathway is used by dopamine via activation of the D1 receptor. Activation of ERK exclusively via NCS-Rapgef2 upon D1 receptor activation *in cellula* prompted us to examine the role of NCS-Rapgef2 in dopaminergic activation of ERK *in vivo*. The ERK pathway has been shown to be essential for long-term synaptic plasticity and behavioral adaptation implicated in the actions of psychomotor stimulants, by activation of transcription factors and phosphorylation of histone H3 in reward-related brain circuitries (Valjent et al., 2000; Brami-Cherrier et al., 2005; Miller and Marshall, 2005; Valjent et al., 2006). These include, besides the NAc, limbic areas such as amygdala (Lu et al., 2005; Radwanska et al., 2005). D1-dependent ERK activation has previously been ascribed to an indirect mechanism involving DARPP-32-dependent inhibition of ERK dephosphorylation (Walaas et al., 1983; Hemmings et al., 1984; Greengard et al., 1999; Svenningsson et al., 2005; Valjent et al., 2005), as well a direct one via PKA-dependent activation of Rap1 and activation of the Ras GDP-releasing protein Rasgrp 2 (Nagai et al., 2016). Here, we have demonstrated a direct NCS-Rapgef2-dependent mechanism for D1-dependent ERK activation, and that this pathway accounts for ERK activation upon D1 receptor activation, and psychomotor stimulant administration, *in vivo*.

What are the implications of the finding that D1-dependent, and psychomotor stimulant-dependent, activation of ERK in three major brain areas requires signaling through NCS-Rapgef2? This finding allows the direct testing of the exigency of signaling through multiple cAMP sensors on dopamine neurotransmission in these key targets of dopamine function. Notably, NCS-Rapgef2 deletion appears to abrogate ERK signaling, while sparing CREB activation in response to dopaminergic signaling. This implies that parcellation of cAMP signaling to various cAMP sensors, including both NCS-Rapgef2, and CREB, obtains in CNS neurons as it does *in cellula*, providing opportunities to assign distinct dopaminergic cellular functions to these two cAMP-dependent pathways. Penetrance to behavioral phenotypes is an obvious next step in this process, and the work of several laboratories (*vide supra*) predicts a role for NCS-Rapgef2, through ERK activation, in some specific dopamine-dependent behaviors driven both by environmental cues and psychomotor stimulants.

## Synthesis

Reviewing Editor: Kirill Martemyanov, The Scripps Research Institute Decisions are customarily a result of the Reviewing Editor and the peer reviewers coming together and discussing their recommendations until a consensus is reached. When revisions are invited, a fact-based synthesis statement explaining their decision and outlining what is needed to prepare a revision will be listed below.

When discussing you manuscript with the reviewer who originally evaluated your manuscript for Journal of Neuroscience we agreed on the significance and impact of the work presented in the manuscript. There was a consensus that the study presents an important advance in large part because it demonstrates novel signal transduction mechanism operating in endogenous setting. It was felt however, that the points that reviewers brought up during their prior review need to be addressed prior to publication. In summary, there are 2 essential domains attention to which is required for this manuscript to be accepted.

1. The manuscript needs to be modified to address reviewer's points. The plan that was outlined in the rebuttal letter is acceptable but these changes, modifications and additions need to be implemented. In addition to submitting a clean copy, please submit the manuscript with the track changes enabled - this would greatly facilitate the evaluation.

2. It was felt that one critical experiment missing from the report, but important for validating the conclusions is a specificity control, i.e. demonstration that Rapgef2 KO does not compromise upstream D1R signaling. This line was originally suggested by reviewer 1 and in my evaluation of the study I agree with this point. This control could entail simply analyzing D1R induced cAMP changes, GTPgS binding, looking at pCREB or anything else that reports intact signaling through D1R independent from Rapgef2. It would need to be done in endogenous striatal neurons and not cell lines.
