# Supplementary figures and images for: NCS-Rapgef2, the Protein Product of the Neuronal Rapgef2 Gene, Is a Specific Activator of D1 Dopamine Receptor-Dependent ERK Phosphorylation in Mouse Brain
Source: eNeuro. 2017 Sep 25;4(5):ENEURO.0248-17.2017. doi: 10.1523/ENEURO.0248-17.2017 (PMC5611689; doi:10.1523/ENEURO.0248-17.2017)

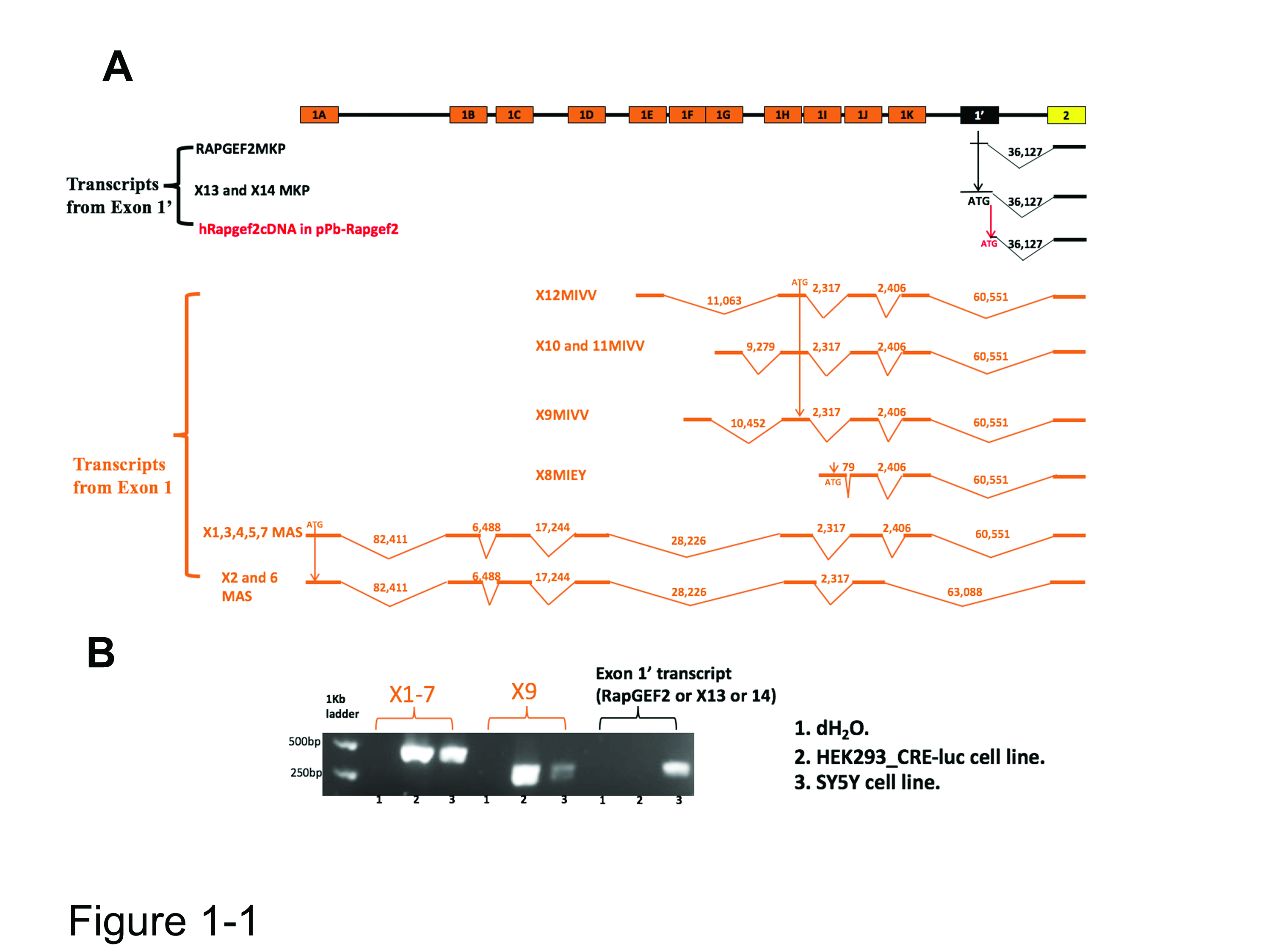

Supplement: Figure 1-1 [file enu005172411so8.tif]

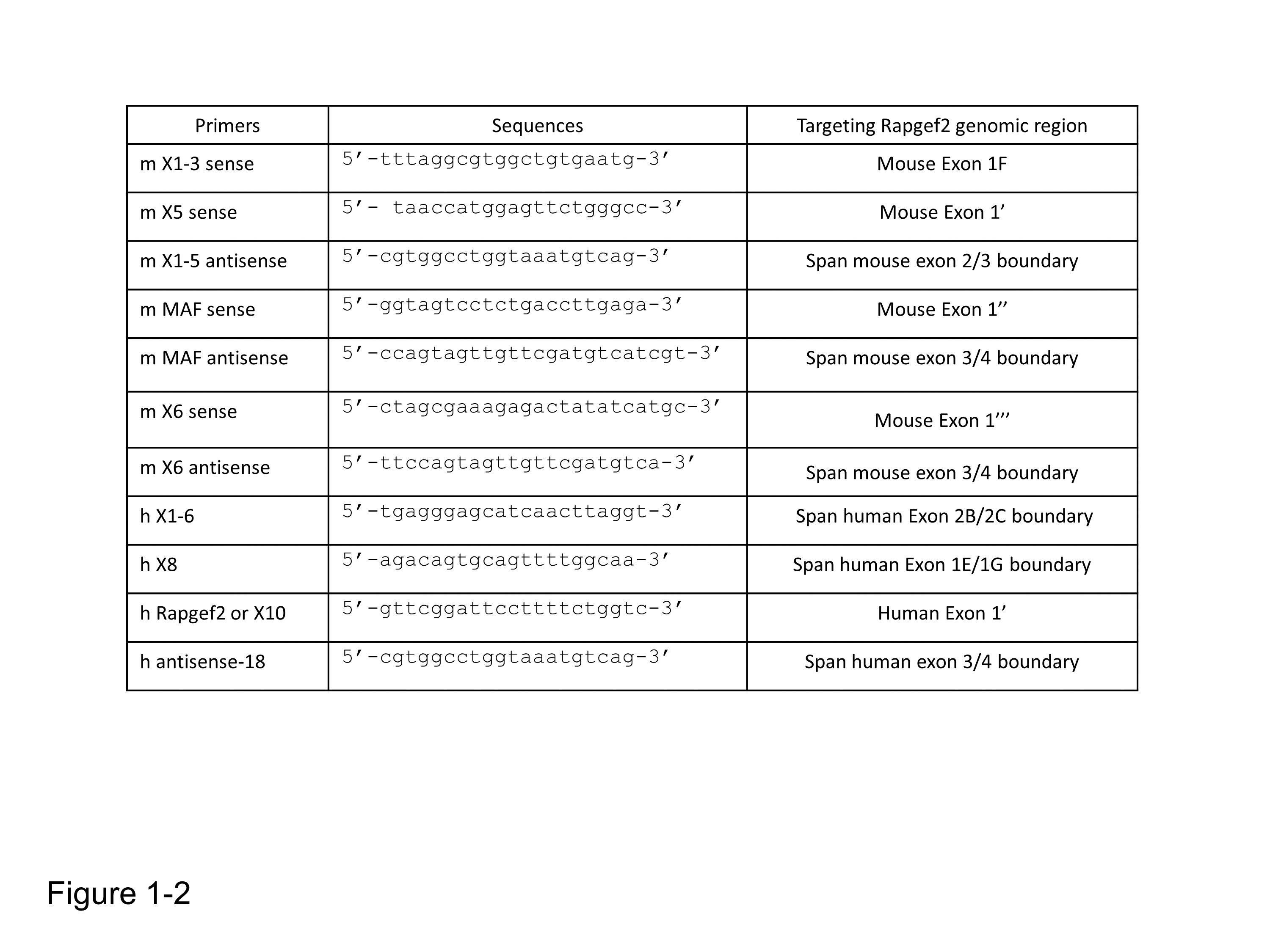

Supplement: Figure 1-2 [file enu005172411so9.tif]

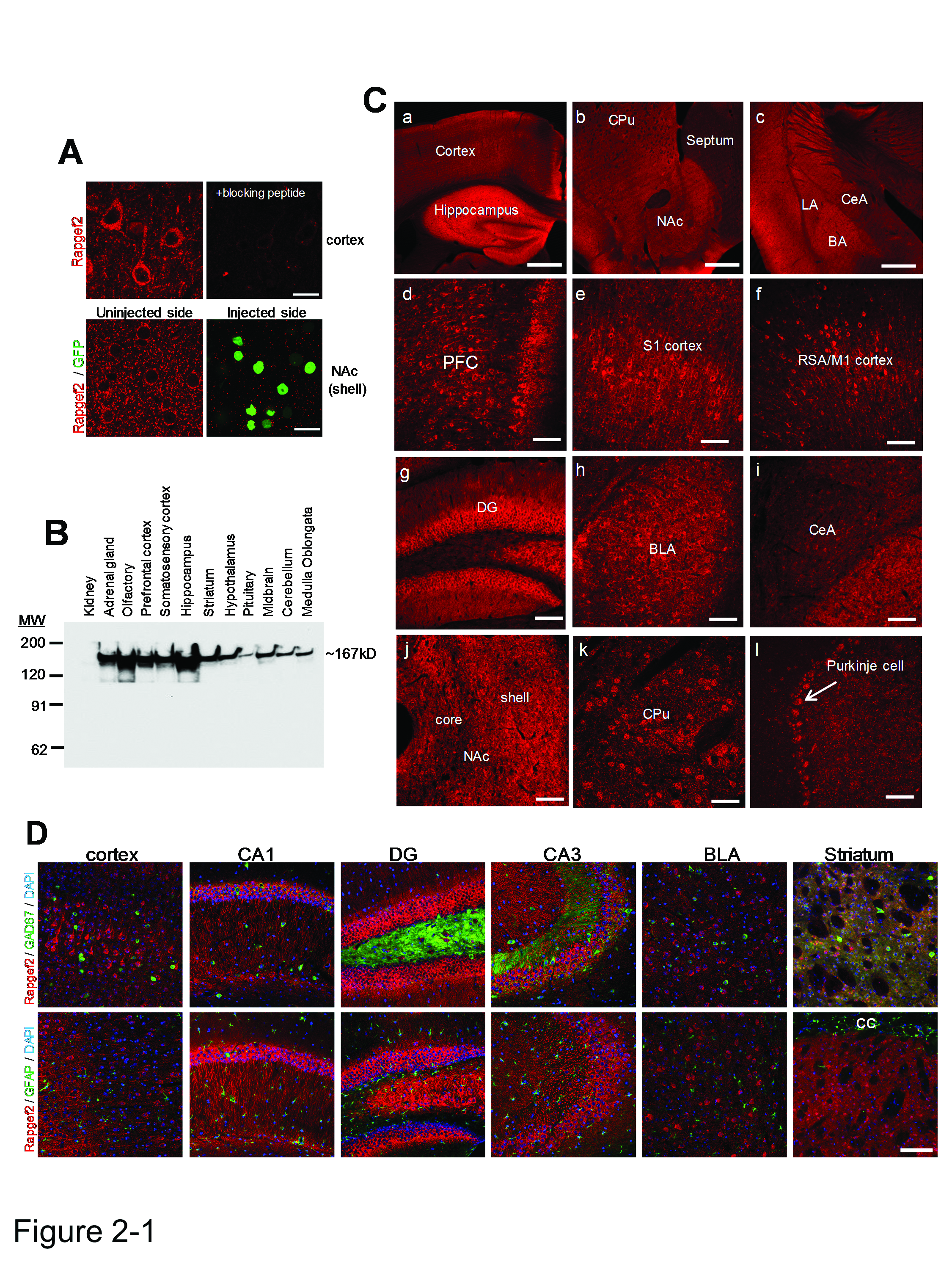

Supplement: Figure 2-1 [file enu005172411so10.tif]
